# A Narrative Review on Oral Microbiome Dysbiosis in Benign and Malignant Oral Lesions: Mechanistic Insights and Clinical Implications

**DOI:** 10.7759/cureus.102131

**Published:** 2026-01-23

**Authors:** Laresh N Mistry, Sayem A Mulla, Amit Patil, Shreyas Shah, Sindhu P Pulaskar

**Affiliations:** 1 Pedodontics and Preventive Dentistry, Bharati Vidyapeeth (Deemed to be University) Dental College and Hospital, Navi Mumbai, IND; 2 Dentistry, Dentist and Independent Researcher, Kalyan, IND; 3 Conservative Dentistry and Endodontics, Bharati Vidyapeeth (Deemed to be University) Dental College and Hospital, Navi Mumbai, IND; 4 Oral Medicine and Radiology, Bharati Vidyapeeth (Deemed to be University) Dental College and Hospital, Navi Mumbai, IND

**Keywords:** microbial dysbiosis, microbiota of oral lesions, oral and maxillofacial pathology, oral microbiology, oral microbiome

## Abstract

Maintaining oral and systemic health depends heavily on the dynamic and varied microbial environment found in the human mouth cavity. There is growing evidence that the oral microbiota plays a role in the pathophysiology of both benign and malignant oral cancers by producing carcinogenic chemicals, modifying host immune responses, and causing chronic inflammation. Malignant tumors like oral squamous cell carcinoma (OSCC) show profound dysbiosis marked by enrichment of pathogenic taxa such as *Porphyromonas gingivalis*, *Prevotella intermedia*, and *Fusobacterium nucleatum*, whereas benign lesions like papillomas, fibromas, and odontogenic tumors show localized microbial alterations. This review examines the molecular processes connecting microbial dysbiosis to carcinogenesis, summarizes current understanding of the oral microbiome across several oral cancers, and emphasizes potential diagnostic, prognostic, and therapeutic applications.

## Introduction and background

The oral microbiome, a complex and varied microbial community found in the mouth cavity, consists of more than 700 different species of bacteria as well as fungi, viruses, archaea, and protozoa. Oral health depends on the symbiotic equilibrium of this ecosystem being maintained. A condition called dysbiosis, which is connected to a number of oral illnesses, such as periodontal disease and dental cavities, arises when this equilibrium is upset [1]. Dysbiosis is a term used to refer to an imbalance in the microbial community's normality, often disrupting host homeostasis, which in the end contributes to the development of any disease. This dysbiosis is also increasingly being linked to the emergence of cancer and systemic diseases. Oral squamous cell carcinoma (OSCC) is one of the most common diseases worldwide, and both benign and malignant oral tumors are serious therapeutic issues [2].

The oral microbiome is increasingly understood to be an active regulator of cancer development rather than a passive dweller. According to research, some microbial species or modifications in the makeup of the community might affect important oncogenic processes. These include encouraging unchecked cell division, allowing tumor cells to evade the immune system, encouraging the development of new blood vessels (angiogenesis) to nourish the tumor, and aiding in the spreading of cancer to other locations [3]. Chronic inflammation, toxins, and metabolites produced by microbes are some of the factors that might produce a pro-tumorigenic microenvironment. The microbiome's potential as a new therapeutic target and a diagnostic marker for oral cancers is highlighted by its active function [4].

The function of the oral microbiota in oral cancers has broad clinical and biological ramifications. From a biological perspective, developing tailored treatments may result from knowing the precise mechanisms via which bacteria affect oncogenesis. Interventions could target microbial-driven inflammatory pathways, eradicate certain oncogenic species, or restore a healthy microbiome [5]. Clinically, the microbial composition of a person's oral cavity may be a biomarker for early oral tumor diagnosis or prognostication [6].

Smoking, alcohol abuse, and long-term stress are important lifestyle variables that have been shown to alter the bacterial makeup of the oral and gastrointestinal microbiome, which in turn affects the risk of cancer. Alcohol changes the permeability of the mucosa and encourages the growth of harmful bacteria that can produce acetaldehyde, a known carcinogen that damages DNA [7]. Due to their pro-inflammatory and immunosuppressive properties, periodontopathogens like *Porphyromonas gingivalis* and *Fusobacterium nucleatum* are linked to oral, esophageal, and colorectal cancers. Smoking introduces toxic substances that upset the oral microbial balance [8]. By changing cortisol-mediated immune responses and promoting the development of opportunistic microorganisms associated with systemic inflammation and tumor progression, chronic stress worsens dysbiosis. Together, these elements provide a microenvironment that is favorable to the development of cancer by increasing genotoxicity, inflammation, and compromised host immunity through microbial changes [9].

In order to improve diagnosis, treatment, and results for patients with both benign and malignant oral cancers, this narrative review highlights the need for more research to convert these discoveries into useful therapeutic applications.

## Review

Methodology

Major scientific databases, such as PubMed, Scopus, Web of Science, and Google Scholar, were methodically searched for English-language literature up until 2025 in order to undertake this narrative review. Oral microbiome, oral cancers, oral benign tumors, microbial dysbiosis, OSCC, odontogenic tumors, Candida, microbial metabolites, and microbiome and carcinogenesis were among the keywords utilized. By cross-referencing pertinent bibliographies, further articles were found. To get a thorough knowledge of microbial interactions in benign and malignant oral cancers, studies including human participants, animal models, and in-vitro investigations were included. Studies with no microbiome-related results, editorials, and non-scientific publications were not included. In order to emphasize mechanistic insights, clinical significance, and new translational uses of the oral microbiota in cancer, the collected material was summarized thematically. A total of 43 articles were included in this.

Oral microbiome in benign tumours

Oral Papilloma

Despite being benign, oral squamous papillomas offer an intriguing illustration of how the oral microbiota might contribute to virus-driven illnesses. Although a human papillomavirus (HPV) infection is the main cause of these papillomas, there is mounting evidence that the microbial environment is more than simply a passive bystander [10]. Dysbiosis, or specific microbial imbalances, might be the cause of a prolonged viral infection. People with these lesions have higher levels of several anaerobic bacterial species in their oral cavities, including *Prevotella* and *Fusobacterium* [11]. By generating toxins and compounds that can harm host cells and inhibit the local immune response, these bacteria provide an environment that is conducive to inflammation. The body may not be able to adequately eradicate the HPV infection due to this persistent inflammation and compromised immune system, which would support the formation of the papilloma and the proliferation of epithelial cells [12].

This combination has important biological and clinical ramifications. The existence of these opportunistic pathogens might be seen from a biological standpoint as a "second hit" that enhances the viral "first hit." For instance, *Fusobacterium* is known to possess adhesion molecules that have the ability to attach to host cells, which may let other microbes infiltrate and exacerbate inflammation [13]. On the other hand, *Prevotella* spp. are known to generate inflammatory cytokines. This knowledge paves the way for novel therapeutic and diagnostic approaches in the clinical setting. In addition to focusing on the HPV virus, controlling the microbial dysbiosis possibly with probiotics or targeted antimicrobial treatments may be a useful strategy to stop the growth of papillomas or help them to go away [14]. By treating both the microbial and viral components, this two-pronged strategy may enhance patient outcomes for this prevalent oral lesion (Table 1).

**Table 1:** Microbial associations seen in oral benign tumors

Tumor type	Dominant/altered microbial species	Possible mechanistic role
Oral papilloma	Prevotella spp., Fusobacterium spp.	In synergism with human papillomavirus (HPV), sustains inflammation & epithelial dysplasia
Fibroma (reactive lesions)	Streptococcus spp., Actinomyces spp.	Promotes chronic inflammation and perpetuate stromal proliferation
Odontogenic tumors	Streptococcus anginosus	Production of carcinogenic metabolites, epithelial interactions and tumor expansion

Fibromas and Other Reactive Lesions

Fibromas and inflammatory hyperplasias can develop as a result of persistent irritation of the oral mucosa. Emerging evidence indicates that microbial colonization has a crucial role in maintaining the inflammatory cycle, even though these disorders are predominantly a tissue reaction to physical damage, such as biting a cheek or poorly fitting dentures. A wound or ulcer is formed when the mucosal barrier is breached by the first physical irritation [15]. The exposed connective tissue can be colonized by opportunistic bacteria, especially facultative anaerobes such as *Streptococcus* spp. and *Actinomyces* spp., thanks to this breach. These bacteria create a variety of virulence factors, including poisons and enzymes, which directly activate the host's immune cells because they thrive in the low oxygen environment of the ulcerated surface [16]. This sets off a protracted inflammatory reaction that is marked by the production of growth factors, chemokines, and pro-inflammatory cytokines as well as the infiltration of immune cells. In addition to impeding wound healing, this ongoing immune activation produces a feedback loop in which the ongoing inflammation encourages more tissue and bacterial growth [17].

Fibroblasts, the cells that make connective tissue, are stimulated to proliferate excessively by the ongoing presence of these bacterial populations and their byproducts. Fibroblast activity is strongly stimulated by pro-inflammatory cytokines generated by immune cells, especially transforming growth factor-beta (TGF-β). As a result, too much collagen and other extracellular matrix components are deposited, which eventually causes a fibroma, a benign development of fibrous connective tissue that resembles a tumor [18]. Similarly, when the body tries to fix the continuous irritation, the tissue remodeling and inflammation lead to inflammatory hyperplasia, which is an increase in the number of cells in the tissue. The intricate interaction between microbial causes and physical damage in the pathophysiology of these frequent oral lesions is highlighted by this vicious cycle of irritation, bacterial colonization, chronic inflammation, and stromal growth [19]. In order to properly treat these disorders, it is necessary to manage the accompanying bacterial burden in addition to eliminating the cause of the physical irritation [20].

Odontogenic Tumors

Ameloblastomas and other odontogenic tumors have microbial communities that are active players in the tumor microenvironment rather than being passive residents. It is especially significant when certain bacteria, such as *Streptococcus anginosus*, are found in the cystic fluids and surrounding tissues of these tumors. This bacterium belongs to the* Streptococcus milleri* group, which is well-known for its propensity to cause abscesses and its link to a number of cancers, including head and neck and gastrointestinal disorders [21]. The metabolic metabolites of *S. anginosus* and other bacteria may function as pro-inflammatory and pro-proliferative signals in odontogenic tumors. For instance, some microbial metabolites, such as butyrate and other short-chain fatty acids (SCFAs), might affect the signaling pathways of host cells. Although butyrate is usually thought to be good for the stomach, its effects might vary depending on the situation [22]. These microbial metabolites may promote the synthesis of growth factors and cytokines, including interleukins and tumor necrosis factor-alpha (TNF-α), in the tumor microenvironment. This results in a persistent inflammatory condition that may encourage the growth of odontogenic epithelial cells and increase the tumor's local invasiveness. Tumor growth may also be aided by bacterial enzymes breaking down the extracellular matrix [23].

Complex host-microbe interactions are involved in the specific processes by which these microbial fingerprints affect the growth of tumors. Bacterial components such as lipopolysaccharides (LPS) might influence the innate immune response of the odontogenic epithelial cells, which are the main constituent of these tumors. This contact can set off a series of events that activate nuclear factor-kappa B (NF-κB) via activating Toll-like receptors (TLRs). NF-κB is a master regulator of genes related to cell survival, proliferation, and inflammation [24]. Persistent microbial stimulation can cause this system to become chronically activated, which can foster tumor development and neoplastic transformation. Moreover, microbial biofilms in the cystic lesions could offer a safe haven for the bacteria and tumor cells, insulating them from the host's immune monitoring and treatment. In order to break this pathological feedback loop, the microbial community and the odontogenic epithelium have a synergistic relationship that opens up a possible new therapeutic approach. One such approach is the use of targeted antimicrobial agents in combination with conventional medical and surgical treatments [25].

Oral microbiome in malignant tumors

Oral squamous cell carcinoma (OSCC)

OSCC grows and spreads in large part due to microbial dysbiosis, especially the enrichment of certain pathogens. This connection has a variety of pathogenic processes. One important mechanism is chronic inflammation, where pro-inflammatory signaling cascades, particularly the NF-κB and Signal transducer and activator of transcription 3 (STAT3) pathways, are activated by recurring bacterial infections caused by pathogens such as *P. gingivalis *and *Treponema denticola* [26]. A microenvironment that promotes unchecked cell proliferation and inhibits apoptosis two characteristics of cancer is produced by this activation [27].

These bacteria produce toxic chemicals that directly contribute to carcinogenesis in addition to causing inflammation. *P. gingivalis* is one of the oral bacteria that may convert alcohol into the Group 1 carcinogen acetaldehyde. These microbes also produce reactive oxygen species (ROS), which have the ability to directly damage DNA and result in genetic changes that encourage the development of cancer [28]. The danger of malignant transformation of oral cells is greatly increased by the combined action of various chemical agents [29].

These microorganisms not only directly produce carcinogens but also influence the host's immune system and promote the growth of tumors. By suppressing the function of natural killer (NK) cells and fostering an environment that is more conducive to tumor development, *F. nucleatum* is especially skilled at immune evasion. Proteases produced by pathogenic bacteria also break down the extracellular matrix (ECM), which is essential for the epithelial-mesenchymal transition (EMT). These bacteria help tumor cells invade and spread to different areas of the body by dissolving the structural barriers [30]. The significance of microbial dysbiosis in fostering OSCC carcinogenesis and progression is highlighted by these combined effects [31].

Oral Verrucous Carcinoma

The pathophysiology of low-grade forms of OSCC is significantly influenced by dysbiosis in the oral microbiome, namely the enrichment of anaerobic bacteria and *Candida albicans*. A microenvironment that is favorable to neoplastic transformation is produced by this microbial imbalance, which is frequently connected to long-term discomfort from things like poorly fitting dentures, sharp teeth, or persistent chewing behaviors. In these circumstances, the commensal yeast *C. albicans *may change into a pathogenic hyphal form [32]. It is well recognized that this kind invades epithelial tissue, causing inflammation and upsetting the typical cellular structure. Additionally, the yeast may convert dietary nitrates and nitrites into N-nitroso compounds, a family of strong carcinogens, in conjunction with certain anaerobic bacteria. Enzymes that are extremely active in the dysbiotic microbiota, such as nitrosating enzymes and nitrate reductases, are involved in this process. The ongoing presence of these microorganisms and their metabolic activities expose the oral mucosa to a steady stream of genotoxic chemicals, raising the possibility of DNA damage and mutations that cause OSCC to start and spread [33].

Beyond just producing nitrosamines, *C. albicans* also plays a co-carcinogenic function. The release of many pro-inflammatory cytokines, including TNF-α, IL-6, and IL-1β, mediates the chronic inflammatory response brought on by its colonization and invasion. This ongoing inflammation encourages angiogenesis, cell division, and apoptosis avoidance, all of which contribute to a pro-tumorigenic environment. Furthermore, the fermentation of carbohydrates by *C. albicans *can result in the production of acetaldehyde, a recognized carcinogen that can further harm DNA [34]. This impact may be made worse by the interaction of *C. albicans* with anaerobic bacteria such *P. gingivalis* and *F. nucleatum*. In addition to contributing to the dysbiotic condition and producing inflammatory byproducts, these bacteria can intensify the carcinogenic processes by generating a positive feedback loop (Table 2).

**Table 2:** Microbial dysbiosis in malignant oral tumors NF-κB:  nuclear factor-kappa B; STAT3: Signal Transducer and Activator of Transcription 3; ROS: reactive oxygen species; EMT: epithelial-mesenchymal transition.

Malignant tumour type	Microorganisms	Mechanism
Oral squamous cell carcinoma (OSCC)	Fusobacterium nucleatum, Porphyromonas gingivalis, Prevotella intermedia, Treponema denticola	Chronic inflammation (NF-κB, STAT3), immune evasion, carcinogen production (acetaldehyde, ROS), invasion/EMT
Verrucous carcinoma	Candida albicans, anaerobic bacteria	Nitrosamine production, chronic irritation, co-carcinogens
Salivary gland carcinomas	Neisseria spp., Streptococcus mitis	Altered salivary microbiome, possible metabolic modulation

Through direct genotoxicity and the establishment of a pro-inflammatory, pro-proliferative microenvironment, these microbial agents collectively have a strong correlation with the development of low-grade OSCC, a disease that is frequently characterized by long-term chronic irritation and progresses slowly [35].

Salivary Gland Tumors

A complicated functional significance that may affect tumor behavior and development is suggested by the increased presence of certain bacteria, such as *Neisseria *and *Streptococcus mitis*, in the salivary microbiome of patients with adenoid cystic carcinoma and mucoepidermoid carcinoma. Even though both genera are prevalent commensals in the healthy oral cavity, their functions can change from symbiotic to harmful if they are overrepresented in a dysbiotic condition [36]. Certain strains of *Neisseria*, for example, have the ability to transform ethanol from alcoholic beverages into acetaldehyde, a recognized carcinogen, through the activity of alcohol dehydrogenase. By increasing local DNA damage and encouraging cellular proliferation, the increased presence of these strains may contribute to a pro-tumorigenic milieu, which might hasten the growth and invasiveness of these carcinomas [37]. On the other hand, studies have revealed that certain commensal strains of *Neisseria* may also have a "probiotic-like" effect, suppressing pro-inflammatory responses and controlling genome stability to inhibit oral squamous cell carcinoma cells. This suggests that their dual function is probably strain-specific and context-dependent [38].

*S. mitis*'s functional involvement in oral tumors is equally complex and needs more research. Despite being widely regarded as a helpful colonizer, some research has revealed that it can limit the growth of oral cancer cells by causing cell cycle arrest. This points to a possible anti-tumorigenic function, perhaps by producing certain metabolites or structural elements that prevent the growth of cancer cells [39]. However, some oral streptococci, such as *S. mitis*, have the ability to alter the host's immune response in ways that might either encourage or prevent the formation of tumors. The host's immune system, the particular genetic and molecular pathways of the tumor, and the general makeup of the salivary microbiome all likely affect how these conflicting roles are balanced. Novel treatment approaches and diagnostic indicators, such as employing certain bacterial strains or their metabolites to stop the spread of cancer, may be made possible by an understanding of these complex microbial-host interactions [40].

Crosstalk between oral microbiome and the host in tumorigenesis

Through a number of interrelated processes, the oral microbiota can stimulate the formation of tumors. First, long-term exposure to microorganisms activates the NOD-like receptor (NLR) and TLR pathways, which in turn sets off inflammatory signals [41]. A microenvironment that is favorable to the growth of cancer cells is produced by this ongoing inflammation. Second, the microbiome affects metabolic pathways; microbial byproducts such as acetaldehyde, hydrogen sulfide, and short-chain fatty acids can directly change cell signaling in the epithelial tissue, promoting the growth of tumors [42]. Last but not least, some infections cause modifications to histones and DNA methylation, which can result in the neoplastic transformation of healthy cells and contribute to epigenetic modulation [43].

Clinical and translational implications

The therapy of OSCC is significantly impacted by the microbiota. Through microbiome analysis, diagnostic indicators from tissue and saliva samples can aid in the early identification of OSCC and its separation from benign lesions. *F. nucleatum* abundance has been associated with a worse prognosis and increased recurrence rates as a prognostic indicator. The microbiome also has therapeutic potential; in addition to conventional treatments, methods such as probiotics, antimicrobial peptides, and other microbiome modification approaches may be employed. Lastly, a more individualized strategy to treating OSCC may be possible with the integration of microbiome signatures into molecular profiling, which is essential for precision oncology.

Limitations

This narrative review has a number of limitations, despite the increasing interest in the function of oral microbiome dysbiosis in benign and malignant oral diseases. The majority of the information currently available comes from cross-sectional and observational research, which limit the ability to draw conclusions about the causal relationship between microbial changes and the advancement of illness. Significant variation in sample types, sequencing platforms, bioinformatic analysis, and study design restricts cross-study comparability and might lead to conflicting results. Furthermore, there is inconsistent control over confounding variables such alcohol and tobacco use, dental hygiene habits, systemic health, and regional variance. The translation of existing discoveries into conclusive therapeutic applications is further restricted by the dynamic nature of the oral microbiome and the scarcity of longitudinal and functional investigations.

Future implications

Even with encouraging results, it is still difficult to determine if microbiota and oral cancers are causally related or not. It is necessary to do extensive longitudinal investigations that use multi-omics techniques such host transcriptomics, metabolomics, and metagenomics. To guarantee repeatability, sampling and sequencing techniques must be standardized. Furthermore, thorough clinical validation is necessary for therapeutic approaches that target the oral microbiota. It is also empirical to identify thresholds of each bacterium in each tumor (benign or malignant). This can be done by using color-coded (bacteria-coded) strip indicators or salivary biomarkers. There is also scope for the development of a novel cancer risk assessment using the levels of bacteria similar to caries risk assessment [44].

## Conclusions

The oral microbiota is a significant but little-studied component in the pathophysiology of both benign and malignant oral diseases. In contrast to benign lesions, which seem to show more localized and less disruptive microbial changes, current evidence consistently shows marked microbial dysbiosis in malignant tumors like OSCC, where altered microbial profiles are linked to inflammation, genomic instability, and tumor-promoting pathways. The clinical translation of microbiome research, particularly its use in prognosis, early diagnosis, and microbiome-targeted therapeutics, is still primarily prospective and has to be validated by longitudinal and mechanistic investigations, despite the growing strength of these relationships.
